# Deletion and low expression of *NFKBIA* are associated with poor prognosis in lower-grade glioma patients

**DOI:** 10.1038/srep24160

**Published:** 2016-04-07

**Authors:** Gabriela Sarti Kinker, Andrew Maltez Thomas, Vinicius Jardim Carvalho, Felipe Prata Lima, André Fujita

**Affiliations:** 1 Department of Physiology, Institute of Bioscience, University of São Paulo, São Paulo, Brazil; 2 Department of Biochemistry, Institute of Chemistry, University of São Paulo, São Paulo, Brazil; 3 Bioinformatics Graduate Program,University of São Paulo, São Paulo, Brazil; 4 Medical Genomics Laboratory, International Research Center, AC Camargo Cancer Center, São Paulo, Brazil; 5 Department of Botany, Institute of Bioscience, University of São Paulo, São Paulo, Brazil; 6 Federal Institute of Alagoas, Alagoas, Brazil; 7 Department of Computer Science, Institute of Mathematics and Statistics, University of São Paulo, São Paulo, Brazil

## Abstract

Lower-grade gliomas (LGGs), which are uniformly fatal in young adults, are classified as grades II-III tumors according to their histological features. The NFκB transcription factor, a crucial player in cancer initiation and progression, is inactivated in the cytoplasm by inhibitory proteins (IκBs) that have been shown to exert tumor-suppressor activity. Therefore, using The Cancer Genome Atlas copy number alteration and RNA-Seq data from 398 patients, we evaluated the association between the expression and dosage of *NFKBIA*, which encodes IκBα, and the overall malignancy of LGGs. Deletion and low expression of *NFKBIA* were associated with enhanced tumor aggressiveness and poor prognosis in LGGs. Accordingly, the dosage and expression of *NFKBIA* were independent prognostic factors for 5-year survival (dosage: *P* = 0.016; expression: *P* = 0.002) and 5-year recurrence-free survival (dosage: *P* = 0.009; expression: *P* = 0.005). Moreover, gene set enrichment analyses and co-expression network analyses indicated a role for *NFKBIA* in the negative regulation of cell proliferation, possibly through the modulation of downstream NFκB activation. Overall, the present findings reveal the prognostic value of *NFKBIA* in LGGs, reinforcing the relevance of NFκB signaling in the development and progression of gliomas.

Lower-grade gliomas (LGGs), which are uniformly fatal in young adults, are infiltrative brain tumors that include astrocytomas, oligoastrocytomas and oligodendrogliomas^1^. The World Health Organization (WHO) classifies these tumors as grades II-III, primarily based on histological features such as mitotic activity, cellularity, nuclear atypia, microvascular proliferation, and necrosis^2^. In addition to traditional morphological histopathology, detailed molecular classification of gliomas also contributes to the WHO grading schemes and will be incorporated into a new integrated diagnosis scheme^3^,^4^,^5^. In this sense, molecular pathology will contribute to the stratification of patients in treatment-specific subgroups, which will lead to the development of more personalized and biologically grounded therapies^6^,^7^.

The NFκB family of transcription factors has an essential role in many biological processes, such as inflammation, innate immunity, cell proliferation and apoptosis^8^. Additionally, aberrant activation of NFκB is increasingly recognized as a crucial factor in cancer initiation and progression^9^. All five members of this protein family (p65, p100/p50, p102/p52, c-Rel and RelB) share a Rel homology domain (RHD), which mediates their dimerization and DNA binding^10^. In most quiescent cells, NFκB dimers remain inactive in the cytoplasm, due to their interaction with inhibitory proteins of the IκB family. The IκBs are characterized by ankyrin repeats, which interact with the RHDs of NFκB proteins, thereby making them transcriptionally inactive^11^. The canonical NFκB pathway is typically triggered by pro-inflammatory cytokines and genotoxic stress, leading to the phosphorylation of IκBα and release of NFκB dimers, mainly p50:p65. NFκB can then translocate to the nucleus and activate the expression of target genes involved in the control of inflammation, cell proliferation, apoptosis, migration and angiogenesis^12^.

NFκB activity in gliomas is significantly higher than in normal brain tissues and phospho-IκBα protein levels have been shown to negatively correlate with tumor grade^13^,^14^,^15^. Additionally, recent studies have revealed that *NFKBIA*, which encodes IκBα, is deleted in approximately 25% of grade IV gliomas (glioblastomas), the most aggressive primary brain tumors^16^. Interestingly, after restoring *NFKBIA* expression in cells cultured from tumors harboring an *NFKBIA* deletion, the malignant phenotype was attenuated and an increase in chemotherapy sensitivity was observed. More importantly, patients with tumors harboring a deletion or low expression of *NFKBIA* demonstrated decreased survival^16^. Accordingly, treatment with nanoparticles loaded with recombinant IκBα and curcumin, a natural polyphenol that inhibits the phosphorylation of IκBα, has been shown to decrease the expression of NFκB target genes such as *CCND1, CCNE1, BCL2L1* and *COX2*, thereby inducing apoptotic cell death in a glioblastoma cell line^17^.

Given the potential role of *NFKBIA* in glioblastoma development and progression^15^,^16^,^17^, we aimed to investigate, in LGGs, the impact of *NFKBIA* dosage and expression on patient survival, overall malignancy and the downstream activation of NFκB.

## Results

### *NFKBIA* deletion frequency and mRNA expression

**Figure 1:**
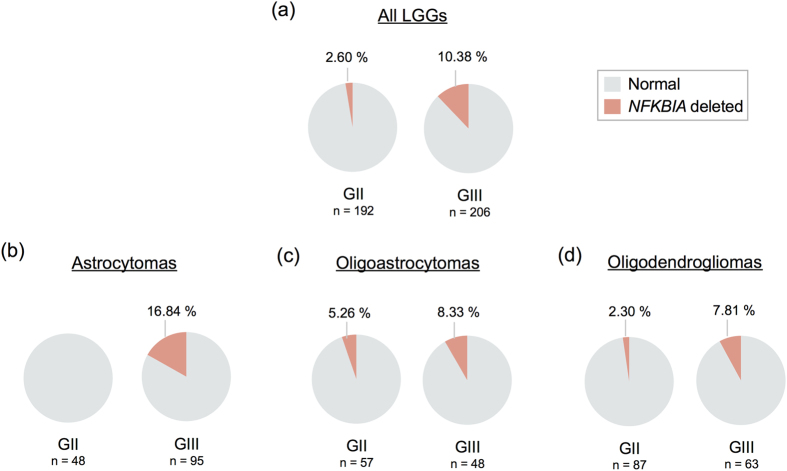
Proportion of *NFKBIA* deletions in lower-grade gliomas (LGGs). *NFKBIA* dosage profile in (**a**) all LGGs, (**b**) astrocytomas, (**c**) oligoastrocytomas and (**d**) oligodendrogliomas according to tumor grade. The CNA magnitudes (x = log_2_ ratio) were classified using simple thresholds: complete deletion (x ≤ −1), deletion (−1 < x ≤ −0.2), and normal (−0.2 < x ≤ 0.2).

**Figure 2:**
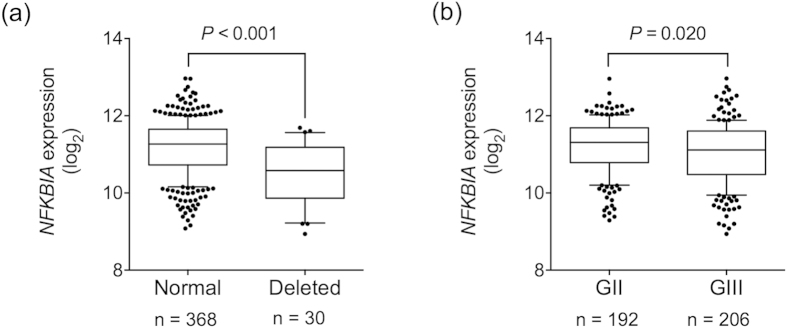
*NFKBIA* expression in lower-grade gliomas (LGGs). RNAseq analysis of *NFKBIA* expression according to (**a**) dosage of *NFKBIA* (normal vs. deleted) and (**b**) tumor grade (GII vs. GIII). Gene expression values were estimated using RSEM. The box extends from the 25th to the 75th percentile, the central bold line shows the median, with whiskers being drawn down to the 10th percentile and up to the 90th. Comparisons were performed using the two-sided Student’s t test.

*NFKBIA* deletion was observed in approximately 7% of LGGs from The Cancer Genome Atlas (TCGA) cohort. The deletion was more common in grade III than in grade II gliomas (Fig. 1, Table 1), regardless of histologic subtype (Fig. 1). Interestingly, grade III astrocytomas, the most aggressive type of LGG, showed the highest frequency of *NFKBIA* deletion (16.84%, Fig. 1a). Deletions were associated with reduced *NFKBIA* mRNA expression (Fig. 2). Accordingly, grade III gliomas expressed significantly lower levels of *NFKBIA* mRNA compared to grade II gliomas. (Fig. 2, Table 2). Patients with LGGs harboring a deletion of *NFKBIA* were significantly older than those with a normal dosage (Table 1).

### Impact of *NFKBIA* dosage and mRNA expression on patient survival

**Figure 3:**
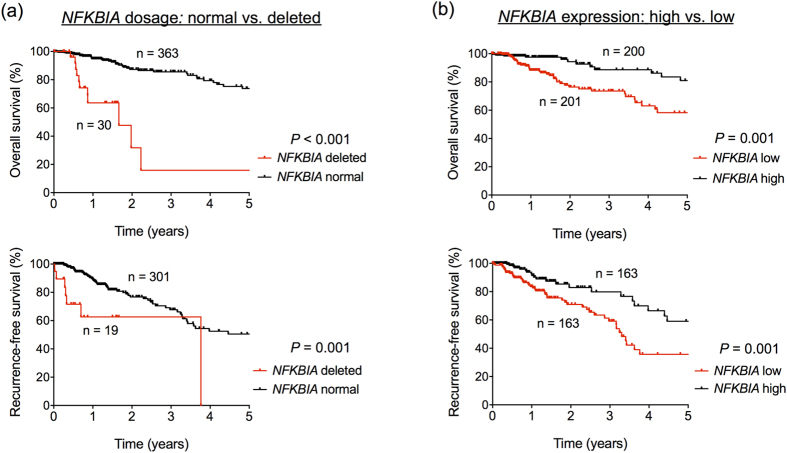
Dosage and expression of *NFKBIA* are prognostic markers in LGGs. Kaplan-Meier analysis of 5-year survival and 5-year recurrence-free survival (RFS) according to (**a**) *NFKBIA* dosage (normal vs. deleted) and (**b**) *NFKBIA* expression (high vs. low). Comparisons were performed using the log-rank test.

When we performed univariate analyses of patient survival using Kaplan-Meier curves and Cox univariate regression models, we found that the dosage and expression of *NFKBIA* were significant prognostic factors in LGGs (Fig. 3 and Table 3). Both deletion and low expression of *NFKBIA* were associated with poor 5-year survival (dosage: HR = 6.54, *P* < 0.001; expression: HR = 0.47, *P* < 0.001, Table 3) and 5-year recurrence-free survival (RFS; dosage: HR = 3.65, *P* = 0.001; expression: HR = 0.58, *P* = 0.001; Table 3). To control for possible confounding factors, we also used a multivariate approach, the Cox multivariate regression model, which allowed us to evaluate survival considering multiple variables simultaneously. After adjusting for age, gender, histological subtype and tumor grade, the dosage and expression of *NFKBIA* remained significant prognostic factors for both 5-year survival (dosage: HR = 2.15, *P* = 0.016; expression: HR = 0.54, *P* = 0.002; Tables 4 and 5) and 5-year RFS (dosage: HR = 3.11 *P* = 0.009; expression: HR = 0.61, *P* = 0.005; Tables 4 and 5). Notably, in the 5-year RFS analyses no other variable was independently associated with prognosis (Tables 4 and 5).

### Effects of *NFKBIA* dosage and mRNA expression on KEGG biological pathways

**Figure 4:**
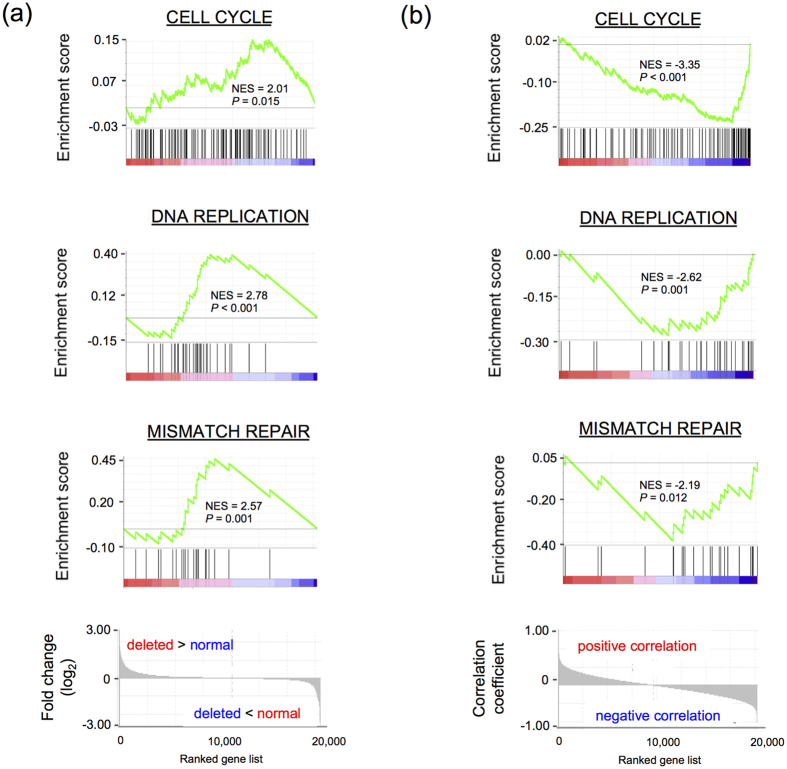
*NFKBIA* has a role in the negative control of cell proliferation. Gene sets involved in the positive control of cell proliferation comprise genes presenting (**a**) increased expression in tumors harboring *NFKBIA* deletions and (**b**) negative correlations with the expression of *NFKBIA*. Normalized enrichment scores (NES) and *P-*values corrected by false discovery rate (FDR) were calculated using GSEA v4.0 and KEGG pathways.

To evaluate the biological relevance of *NFKBIA* dosage and expression in LGGs, we performed gene set enrichment analysis (GSEA) using genes ranked according to i) their differential expression in tumors with *NFKBIA* deletion or ii) their Pearson’s correlation with the expression of *NFKBIA.* Among KEGG pathways overexpressed in tumors harboring a deletion of *NFKBIA*, 32 were significantly enriched (*P* < 0.05 corrected by false discovery rate, FDR; Supplementary Table S1). On the other hand, among KEGG pathways negatively correlated with the expression of *NFKBIA*, 10 were significantly enriched (*P* < 0.05 corrected by FDR; Supplementary Table S2). Interestingly, all three pathways significantly enriched in both analyses, namely “cell cycle,” “DNA replication” and “mismatch repair,” are implicated in the process of cell proliferation (Fig. 4).

### Co-expression analysis of NFκB target genes

**Figure 5:**
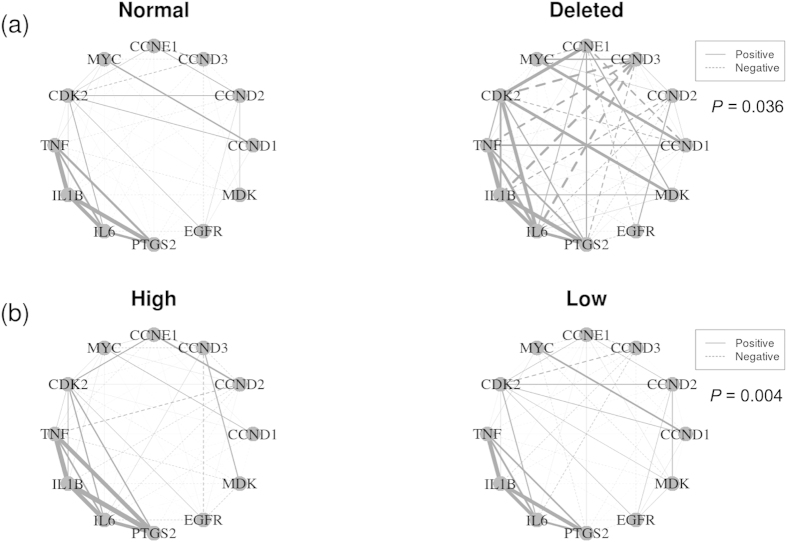
Deletion and low expression of *NFKBIA* alter the co-expression pattern of pro-proliferation NFκB target genes. Undirected and weighted networks were built using (**a**) tumors with normal (left) vs. deleted (right) *NFKBIA* and (**b**) tumors with high (left) vs. low (right) *NFKBIA* expression. Positive correlations are indicated with continuous lines and negative correlations with dashed lines. Edge weights (thicknesses) are proportional to the Spearman’s correlation coefficient between gene pairs. Networks were compared using CoGA software and *P*-values corrected by false discovery rate (FDR) are indicated.

Given that IκBα proteins inhibit the transcriptional activity of p50:p65 NFκB dimers, we sought to investigate if the anti-proliferative role of *NFKBIA,* indicated by the GSEA, was associated with changes in the expression pattern of NFκB target genes. As such, we performed co-expression network analyses using NFκB target genes involved in the positive control of cell proliferation, such as *CCND1, MYC, IL6* and *EGFR*. Interestingly, both deletion and low expression of *NFKBIA* significantly affected the network’s spectral distribution (dosage: *P = *0.036; expression: *P* = 0.004; Fig. 5), showing that pro-proliferation NFκB target genes were differentially co-expressed between phenotypes (deleted vs. normal and high vs. low; Fig. 5).

## Discussion

Previous studies have suggested a correlation between the levels of phospho-IκBα and the grade of gliomas^15^; however, to the best of our knowledge, there is no available data assessing the biological and clinical implications of *NFKBIA* dosage and expression in LGGs. Thus, the present findings demonstrate that deletion and low expression of *NFKBIA* are associated with enhanced tumor aggressiveness and poor prognosis in LGGs. Moreover, our data indicate a role for *NFKBIA* in the negative control of cell proliferation, possibly through inhibition of NFκB transcriptional activity.

The NFκB signal transduction cascade is a multi-component pathway that ultimately controls the expression of genes involved in multiple biological processes^11^. The effect of upstream components of the pathway on the activity of NFκB usually determines the expression pattern of target genes^18^,^19^. The dysregulation of the NFκB pathway at different levels, either by mutations, epigenetic mechanisms or pharmacological means, is involved in many human diseases, especially chronic inflammation, immunodeficiency and cancer^20^,^21^,^22^,^23^. Notably, NFκB is aberrantly activated in tumor cells; however, the mechanisms of activation appear to be complex and vary in different tumor types^9^,^12^. Given that the modulation of NFκB activity has an important role in the prevention and management of cancer, careful evaluation of its complex regulation in different tumors is essential^24^,^25^.

In this study, we demonstrate that *NFKBIA*, which encodes IκBα, a critical negative regulator of NFκB canonical activation, is heterozygously deleted in approximately 7% of LGGs. Additionally, grade III tumors presented a higher frequency of *NFKBIA* deletion, combined with reduced mRNA expression, suggesting an association between *NFKBIA* and overall glioma malignancy. More importantly, the dosage and expression of *NFKBIA* were revealed as grade- and histological subtype-independent prognostic factors for both 5-year survival and 5-year RFS. In both cases, the deletion and low expression of *NFKBIA* were associated with poor prognosis, corroborating the idea that IκB proteins demonstrate tumor suppressor functions^23^,^26^.

When released from IκBα proteins, p50:p65 NFκB dimers can promote cell proliferation by regulating the mRNA expression of cell cycle machinery genes, inflammatory cytokines and growth factors^27^,^28^,^29^,^30^. Accordingly, our data indicate that *NFKBIA* has a role in the negative control of cell proliferation, changing the co-expression pattern of NFκB target genes. In this sense, GSEA revealed that the expression of many genes involved in cell cycle progression was increased in tumors with *NFKBIA* deletion and negatively correlated with the expression of *NFKBIA.* Moreover, co-expression network analyses suggested that deletions and low expression of *NFKBIA* could promote cell proliferation possibly by interfering with the expression pattern of NFκB target genes. Nevertheless, further studies are needed to better understand the mechanistic implications of deletions and low expression of *NFKBIA* in the control of NFκB signaling in LGGs. In particular, it would be relevant to determine which NFκB dimers are more frequently activated in the absence of *NFKBIA*, and if this aberrant activation could contribute to the transcription of pro-tumoral genes and, consequently, to the acquisition of a more malignant phenotype *in vitro* and *in vivo*.

The characterization of molecular markers/profiles of LGGs associated with poor outcomes can lay the biological groundwork for the development of rationally designed targeted therapies to improve patient survival. In this sense, despite the limitations intrinsic to our data, the present findings support a role for *NFKBIA* in the control of LGG malignancy, reinforcing the relevance of NFκB signaling in the development and progression of gliomas^31^. Thus, therapies that stabilize NFκB-IκBα interactions in the cytoplasm might effectively restrain oncogenic signaling, especially in tumors presenting a deletion or low expression of *NFKBIA*.

## Materials and Methods

### TCGA data

We obtained clinical, RNA-Seq (V2), and Copy Number Alteration (CNA) level 3 data from LGGs in TCGA^32^ using the Cancer Genomics Hub portal^33^ and the TCGA-Assembler package^34^. Datasets comprised clinical data from 530 patients, CNA data from 512 patients and RNA-Seq data from 528 patients, the intersection of which consisted of 512 cases for which all three types of data were present. Patients with missing histological grade were excluded from this study, leading to a set of 398 cases, which were used in all the analyses. All data pre-processing was performed using the R software package (http://www.r-project.org).

CNA detection was performed using the Affymetrix (Santa Clara, USA) *Genome-Wide Human SNP Array 6.0* platform, with approximately 1.8 million genetic markers divided into 900,00 SNP and 906,600 CNA detection probes, spread across the human genome. Data processing was performed using GenePattern’s Affymetrix SNP6 Copy Number Inference pipeline. Normalization of CNA values was performed using the circular binary segmentation algorithm^35^. The magnitude of *NFKBIA* CNAs was measured using a simplified version of a previous classification scheme, where tumors were labeled as “complete deletion” when the log_2_ of the normalized CNA value was less than or equal to −1: “deletion,” when the value was between −1 and −0.2, or “normal,” when the value was between −0.2 and 0.2^36^.

RNA sequencing was performed using the *Illumina HiSeq 2000* platform and data processing was performed through the second analysis pipeline (RNASeqV2), using MapSplice^37^ and RSEM^38^ for gene mapping and gene expression quantification, respectively. Tumors were dichotomized, as “low” or “high,” according to *NFKBIA* expression using the median expression value as a cutoff.

### GSEA

All genes from TCGA RNAseq dataset were pre-ranked according to: i) their differential expression (fold change) comparing tumors with normal and deleted *NFKBIA* dosages (median_deleted_/median_normal_), or ii) Pearson’s correlation between their expression and the expression of *NFKBIA*. GSEA was performed using GSEA v4.0^39^ and KEGG pathways^40^. Enrichment scores (ES) were calculated based on a Kolmogorov–Smirnov statistic and tested for significance using 1,000 permutations. ES were further normalized (NES) to account for the size of each gene set. *P*-values corresponding to each NES were corrected for multiple comparisons by the FDR procedure^39^. Adjusted *P*-values < 0.05 were considered statistically significant.

### Co-expression network analysis

To construct co-expression networks, we selected NFκB target genes involved in the positive control of cell proliferation, namely: *CCND1, CCND2, CCND3, CCNE1, CDK2, MYC, TNF, IL1B, IL6, EGFR, MDK, PTGS2*. In these undirected weighted co-expression networks, genes are nodes, while edges represent the pairwise correlations between gene expressions. Edge weights correspond to the Spearman’s correlation coefficient between gene pairs. Networks were visualized using the igraph package^41^ in R (http://www.r-project.org).

We used CoGA software^42^ to compare networks built according to *NFKBIA* dosage (normal vs. deleted) and expression (high vs. low). CoGA identifies structural differences between networks by using graph spectral distribution. The spectrum of a graph is the set of eigenvalues of its adjacency matrix. The spectrum is a general way to describe the structure of a network and can be used to determine if two networks were generated by the same model^43^. We considered that two networks were significantly different by rejecting the null hypothesis of the equality test with an adjusted *P*-value (corrected for multiple comparisons by the FDR procedure) threshold of 5%.

### Statistical Analysis

Two-group comparisons were analyzed using two-sided Student’s t tests. The chi-square test was used to access the association between various categorical clinicopathological characteristics and *NFKBIA* dosage (normal vs. deleted) and expression (high vs. low). We evaluated the impact of *NFKBIA* dosage and expression on both patient overall survival and RFS using Kaplan-Meier curves and the log-rank test^44^ in addition to uni- and multivariate Cox proportional hazard models^45^. HRs, including 95% confidence intervals, were calculated. The survival time was right-censored by 5 years. Statistical analyses were performed with GraphPad Prism 6 and R (http://www.r-project.org). *P*-values < 0.05 were considered statistically significant.

## Additional Information

**How to cite this article**: Kinker, G. S. *et al*. Deletion and low expression of *NFKBIA* are associated with poor prognosis in lower-grade glioma patients. *Sci. Rep.* **6**, 24160; doi: 10.1038/srep24160 (2016).

## Supplementary Material

Supplementary Information

## Figures and Tables

**Table 1:** Clinicopathological features according to the dosage of *NFKBIA*.

Variables	*NFKBIA* dosage		
	Deleted (n = 30)	Normal (n = 368)	*P*-value*
Age, y			
Mean (SD)	53.3 (12)	42.3 (13.2)	<0.001
Gender, %			
Male	50.0	55.6	0.681
Female	50.0	44.4	
Histological subtype, %			
Astrocytoma	53.4	34.6	0.092
Oligoastrocytoma	23.3	26.1	
Oligodendroglioma	23.3	40.3	
Histological grade, %			
II	16.7	50.4	<**0.001**
III	83.3	49.6	

*Two-sided Student’s t-test (continuous variables) or the chi-square test (categorical variable).

**Table 2:** Clinicopathological features according to the expression of *NFKBIA.*

Variables	*NFKBIA* expression		
	Low (n = 208)	High (n = 209)	*P*-value*
Age, y			
Mean (SD)	43.4 (14.2)	42.8 (12.6)	0.671
Gender, %			
Male	47.1	42.6	0.402
Female	52.9	57.4	
Histological subtype, %			
Astrocytoma	39.9	32.0	0.173
Oligoastrocytoma	26.0	25.8	
Oligodendroglioma	34.1	42.2	
Histological grade, %			
II	42.3	53.6	**0.027**
III	57.7	46.4	

*Two-sided Student’s t-test (continuous variables) or the chi-square test (categorical variable).

**Table 3:** Univariate Cox regression analysis of 5-year survival and 5-year recurrence-free survival.

Variables	5-year survival		5-year RFS	
	HR (95% CI)	*P*-value	HR (95% CI)	*P*-value
Age	1.084 (1.06–1.108)	<0.001	1.008 (0.989–1.029)	0.371
Gender				
Female vs. Male	1.011 (0.578–1.769)	0.968	0.824 (0.522–1.302)	0.408
Histological subtype				
Astrocytoma vs. Oligodendroglioma	0.463 (0.244–0.881)	0.019	0.795 (0.462–1.370)	0.409
Astrocytoma vs. Oligoastrocytoma	0.468 (0.224–0.976)	0.042	0.828 (0.455–1.509)	0.539
Histological grade				
II vs. III	1.708 (2.59–11.77)	<**0.001**	1.348 (0.848–2.141)	0.206
*NFKBIA* dosage				
Normal vs. Deleted	6.537 (3.261–13.11)	<**0.001**	3.65 (1.653–8.06)	**0.001**
*NFKBIA* expression	0.470 (0.322–0.685)	<**0.001**	0.584 (0.420–0.813)	**0.001**

RFS, recurrence-free survival. HR, hazard ratio; CI, confidence interval.

**Table 4:** Multivariate Cox regression analysis of 5-year survival and 5-year recurrence-free survival according to the dosage of *NFKBIA*.

Variables	5-year survival		5-year RFS	
	HR (95% CI)	*P*-value	HR (95% CI)	*P*-value
Age	1.084 (1.056–1.112)	<**0.001**	1.013 (0.992–1.034)	0.208
Gender				
Female vs. Male	0.928 (0.520–1.656)	0.802	0.901 (0.542–1.500)	0.690
Histological subtype				
Astrocytoma vs. Oligodendroglioma	0.423 (0.209–0.853)	0.016	0.729 (0.390 – 1.363)	0.322
Astrocytoma vs. Oligoastrocytoma	0.542 (0.250–1.177)	0.122	0.969 (0.509–1.842)	0.923
Histological grade				
II vs. III	2.923 (1.308–6.528)	**0.008**	1.156 (0.669–1.998)	0.602
*NFKBIA* dosage				
Normal vs. Deleted	2.158 (1.018–4.572)	**0.016**	3.111 (1.323–7.318)	**0.009**

RFS, recurrence-free survival. HR, hazard ratio; CI, confidence interval.

**Table 5:** Multivariate Cox regression analysis of 5-year survival and 5-year recurrence-free survival according to the expression of *NFKBIA*.

Variables	5-year survival		5-year RFS	
	HR (95% CI)	*P*-value	HR (95% CI)	*P*-value
Age	1.087 (1.061–1.144)	<**0.001**	1.013 (0.993–1.034)	0.196
Gender				
Female vs. Male	1.057 (0.596–1.875)	0.847	0.902 (0.552–1.474)	0.681
Histological subtype				
Astrocytoma vs. Oligodendroglioma	0.394 (0.200–0.774)	**0.006**	0.694 (0.377–1.277)	0.240
Astrocytoma vs. Oligoastrocytoma	0.480 (0.223–1.031)	0.060	0.842 (0.447–1.583)	0.593
Histological grade				
II vs. III	2.596 (1.159–5.814)	**0.020**	1.129 (0.657–1.939)	0.659
*NFKBIA* expression	0.544 (0.367–0.806)	**0.002**	0.616 (0.437–0.868)	**0.005**

RFS, recurrence-free survival. HR, hazard ratio; CI, confidence interval.
