# Supplementary material for: Deletion and low expression of NFKBIA are associated with poor prognosis in lower-grade glioma patients
Source: Sci Rep. 2016 Apr 7;6:24160. doi: 10.1038/srep24160 (PMC4823696; doi:10.1038/srep24160)
Supplement: Supplementary Information [file srep24160-s1.pdf]

Deletion and low expression of *NFKBIA* are associated with poor prognosis in lower-grade glioma patients

Gabriela Sarti Kinker[1], Andrew Maltez Thomas[2,3,4], Vinicius Jardim Carvalho[3,5], Felipe Prata Lima[2,3,6], André Fujita[7]*

[1] *Department of Physiology, Institute of Bioscience, University of São Paulo, São Paulo, Brazil.*

[2] *Department of Biochemistry, Institute of Chemistry, University of São Paulo, São Paulo, Brazil.*

[3] *Bioinformatics Graduate Program,University of São Paulo, São Paulo, Brazil.*

[4] *Medical Genomics Laboratory, International Research Center, AC Camargo Cancer Center, São Paulo, Brazil.*

[5] *Department of Botany, Institute of Bioscience, University of São Paulo, São Paulo, Brazil.*

[6] *Federal Institute of Alagoas, Alagoas, Brazil.*

[7] *Department of Computer Science, Institute of Mathematics and Statistics, University of São Paulo, São Paulo, Brazil.*

* Corresponding Author: André Fujita, Department of Computer Science, Institute of Mathematics and Statistics, Rua do Matão, 1010, Cidade Universitária, 05508-090 - Sao Paulo, SP - Brazil. Telephone: 55 (11) 30915177. Email: fujita@ime.usp.br.

Supplementary Tables

Table S1. KEGG pathways overrepresented (P < 0.05 corrected by false discovery rate) in *NFKBIA* deleted vs. *NFKBIA* normal lower-grade gliomas.

	Gene Set	Size	Enrichment Score	Normalized Enrichment Score	FDR q-value
1	KEGG_CYTOKINE_CYTOKINE_RECEPTOR_INTERACTION	213	0.32	5.34	<0.001
2	KEGG_INTESTINAL_IMMUNE_NETWORK_FOR_IGA_PRODUCTION	42	0.65	4.89	<0.001
3	KEGG_SYSTEMIC_LUPUS_ERYTHEMATOSUS	101	0.37	4.41	<0.001
4	KEGG_AUTOIMMUNE_THYROID_DISEASE	33	0.62	4.26	<0.001
5	KEGG_GRAFT_VERSUS_HOST_DISEASE	31	0.63	4.26	<0.001
6	KEGG_ASTHMA	23	0.71	4.13	<0.001
7	KEGG_ECM_RECEPTOR_INTERACTION	82	0.38	4.04	<0.001
8	KEGG_COMPLEMENT_AND_COAGULATION_CASCADES	53	0.45	3.96	<0.001
9	KEGG_ALLOGRAFT_REJECTION	31	0.6	3.95	<0.001
10	KEGG_HEMATOPOIETIC_CELL_LINEAGE	76	0.38	3.87	<0.001
11	KEGG_CELL_ADHESION_MOLECULES_CAMS	127	0.25	3.3	<0.001
12	KEGG_TYPE_I_DIABETES_MELLITUS	37	0.46	3.22	<0.001
13	KEGG_LEISHMANIA_INFECTION	67	0.34	3.18	<0.001
14	KEGG_NATURAL_KILLER_CELL_MEDIATED_CYTOTOXICITY	110	0.25	3.01	<0.001
15	KEGG_PRIMARY_IMMUNODEFICIENCY	31	0.44	2.95	<0.001
16	KEGG_JAK_STAT_SIGNALING_PATHWAY	115	0.24	2.91	<0.001
17	KEGG_DNA_REPLICATION	36	0.4	2.78	<0.001
18	KEGG_ANTIGEN_PROCESSING_AND_PRESENTATION	64	0.29	2.67	<0.001
19	KEGG_MISMATCH_REPAIR	23	0.45	2.57	0.001
20	KEGG_FOCAL_ADHESION	195	0.15	2.5	0.001
21	KEGG_VIRAL_MYOCARDITIS	63	0.24	2.27	0.003
22	KEGG_LYSOSOME	118	0.17	2.17	0.006
23	KEGG_N_GLYCAN_BIOSYNTHESIS	46	0.27	2.11	0.008
24	KEGG_GLYCOSAMINOGLYCAN_DEGRADATION	19	0.4	2.06	0.011
25	KEGG_CELL_CYCLE	118	0.16	2.01	0.015
26	KEGG_LEUKOCYTE_TRANSENDOTHELIAL_MIGRATION	108	0.17	2.01	0.015
27	KEGG_HOMOLOGOUS_RECOMBINATION	26	0.32	2.01	0.014
28	KEGG_STARCH_AND_SUCROSE_METABOLISM	31	0.3	1.98	0.017
29	KEGG_NUCLEOTIDE_EXCISION_REPAIR	44	0.24	1.93	0.021
30	KEGG_GLUTATHIONE_METABOLISM	43	0.25	1.93	0.020
31	KEGG_TOLL_LIKE_RECEPTOR_SIGNALING_PATHWAY	88	0.17	1.89	0.024
32	KEGG_PEROXISOME	75	0.18	1.79	0.041

Table S2. KEGG pathways negatively correlated (P < 0.05 corrected by false discovery rate) with the expression of *NFKBIA* in lower-grade gliomas.

	Gene Set	Size	Enrichment Score	Normalized Enrichment Score	FDR q-value
1	KEGG_CELL_CYCLE	118	-0.26	-3.25	<0.001
2	KEGG_OOCYTE_MEIOSIS	112	-0.23	-2.85	<0.001
3	KEGG_NEUROACTIVE_LIGAND_RECEPTOR_INTERACTION	271	-0.14	-2.75	0.001
4	KEGG_AXON_GUIDANCE	129	-0.2	-2.67	0.001
5	KEGG_DNA_REPLICATION	36	-0.37	-2.62	0.001
6	KEGG_UBIQUITIN_MEDIATED_PROTEOLYSIS	134	-0.19	-2.52	0.001
7	KEGG_CALCIUM_SIGNALING_PATHWAY	177	-0.16	-2.48	0.002
8	KEGG_PROGESTERONE_MEDIATED_OOCYTE_MATURATION	85	-0.21	-2.23	0.010
9	KEGG_MISMATCH_REPAIR	23	-0.39	-2.19	0.012
10	KEGG_GAP_JUNCTION	87	-0.18	-2.01	0.034
